# Engineering of chimeric enzymes with expanded tolerance to ionic strength

**DOI:** 10.1128/spectrum.03546-23

**Published:** 2024-05-02

**Authors:** Paweł Mitkowski, Elżbieta Jagielska, Izabela Sabała

**Affiliations:** 1 International Institute of Molecular and Cell Biology in Warsaw, Warsaw, Poland; 2 Mossakowski Medical Research Institute Polish Academy of Sciences, Warsaw, Poland; Suranaree University of Technology, Nakhon Ratchasim, Thailand; Kasetsart University, Bangkok, Thailand

**Keywords:** EnpA, SH3b, M23, bacteriolytic enzymes, chimera, *S. aureus*, *E. faecalis*

## Abstract

Antimicrobial resistance poses a significant global threat, reaching dangerously high levels as reported by the World Health Organization. The emergence and rapid spread of new resistance mechanisms, coupled with the absence of effective treatments in recent decades, have led to thousands of deaths annually from infections caused by drug-resistant microorganisms. Consequently, there is an urgent need for the development of new compounds capable of combating antibiotic-resistant bacteria. A promising class of molecules exhibiting potent bactericidal effects is peptidoglycan hydrolases. Previously, we cloned and characterized the biochemical properties of the M23 catalytic domain of the EnpA (EnpA_CD_) protein from *Enterococcus faecalis*. Unlike other enzymes within the M23 family, EnpA_CD_ demonstrates broad specificity. However, its activity is constrained under low ionic strength conditions. In this study, we present the engineering of three chimeric enzymes comprising EnpA_CD_ fused with three distinct SH3b cell wall-binding domains. These chimeras exhibit enhanced tolerance to environmental conditions and sustained activity in bovine and human serum. Furthermore, our findings demonstrate that the addition of SH3b domains influences the activity of the chimeric enzymes, thereby expanding their potential applications in combating antimicrobial resistance.

**IMPORTANCE:**

These studies demonstrate that the addition of the SH3b-binding domain to the EnpA_CD_ results in generation of chimeras with a broader tolerance to ionic strength and pH values, enabling them to remain active over a wider range of conditions. Such approach offers a relatively straightforward method for obtaining antibacterial enzymes with tailored properties and emphasizes the potential for proteins’ engineering with enhanced functionality, contributing to the ongoing efforts to address antimicrobial resistance effectively.

## INTRODUCTION

Antimicrobial resistance (AMR) is a worldwide problem accelerated by the inappropriate use and overconsumption of antibiotics across human healthcare, agriculture, and industry. The World Health Organization has identified bacterial species, termed “priority pathogens,” that pose the greatest threat to human health. These pathogens, *Enterococcus faecium*, *Staphylococcus aureus*, *Klebsiella pneumoniae*, *Acinetobacter baumannii*, *Pseudomonas aeruginosa,* and *Enterobacter* spp., collectively known as ESKAPE, were accountable for 929,000 deaths attributed to AMR and 3.57 million deaths associated with AMR globally in 2019 (1, 2). Over the past three decades, only two new classes of antibiotics have been approved for human treatment (3). Consequently, there is an urgent need to intensify research efforts aimed at discovering new compounds and innovative strategies to combat bacterial infections effectively.

Peptidoglycan hydrolases (PGHs) represent a highly promising group of molecules with potent bactericidal effects. These enzymes cleave bonds within bacterial cell walls leading to instant death of bacteria (4). Peptidoglycan (PG) serves as the primary polymer in bacterial cell walls, comprising sugar moieties and amino acids that form a mesh-like structure encasing the cells. The sugar chain contains alternating residues of β-(1,4)-linked N-acetylglucosamine and N-acetylmuramic acid. Murein peptides, attached to N-acetylmuramic acid, establish connections between the sugar chains. The composition of PG, particularly its peptide components, is characteristic of specific bacterial genera or species. However, it can undergo alterations based on environmental conditions or the bacterial growth phase (5). For instance, *S. aureus* or *Enterococcus faecalis* contain a murein peptide with the following composition: L-Ala-D-iGlu-L-Lys-D-Ala-D-Ala and the cross-linking peptide: Gly-Gly-Gly-Gly-Gly and L-Ala-L-Ala, respectively (6, 7).

PGHs typically exhibit a modular architecture consisting of well-defined catalytic and binding domains (8). EnpA, originating from bacteriophage and found in the genome of *E. faecalis*, is a large multidomain protein that comprises a phage-related tail protein domain, murein D,D-endopeptidase MepM, murein hydrolase activator NlpD containing a LysM domain, peptidase M23 and lytic transglycosylase-like domain. M23 domains, categorized as zinc metallo-endopeptidases, play a pivotal role in cleaving bonds within PG (9). In our previous studies, we demonstrated that the isolated M23 domain of EnpA (EnpA_CD_) exhibits potent lytic activity against a broad spectrum of bacterial species, including its host *E. faecalis* as well as some staphylococci and streptococci, but its activity is limited to low ionic strength (10).

In multidomain PGHs, catalytic domains are typically accompanied by cell wall-binding domains, which facilitate the enzyme attachment to the cell surface. They belong to various families, with SH3b, LysM, ChBD, and Clp7 being among the most common ones (11). Studies have demonstrated that these domains recognize distinct elements within bacterial cell walls. For instance, the LysM domain binds to glycan chains of PG (12), while SH3b domain, found in enzymes such as lysostaphin and Ale-1, recognizes pentaglycine cross-bridges present in staphylococcal PG (13, 14).

In our study, we have successfully engineered chimeric enzymes comprising EnpA_CD_ and SH3b domains sourced from three distinct staphylococcal PGHs. These chimeras effectively retained the specificity of EnpA_CD_ while demonstrating enhanced activity across an expanded range of ionic strength and pH conditions. With these improved characteristics, the chimeric enzymes hold significant potential for diverse applications as a novel class of nonantibiotic antibacterial agents. Furthermore, our analysis of the three chimeric enzymes, each featuring the same catalytic domain alongside different SH3b domains, has provided valuable insights into additional mechanisms governing interactions between SH3b domains and bacterial cell walls. This investigation sheds light on the diverse functionalities of SH3b domains beyond direct PG binding, further enriching our understanding of the molecular mechanisms underlying bacterial cell lysis and enzyme activity.

## RESULTS

### Chimera design and purification

**FIG 1:**
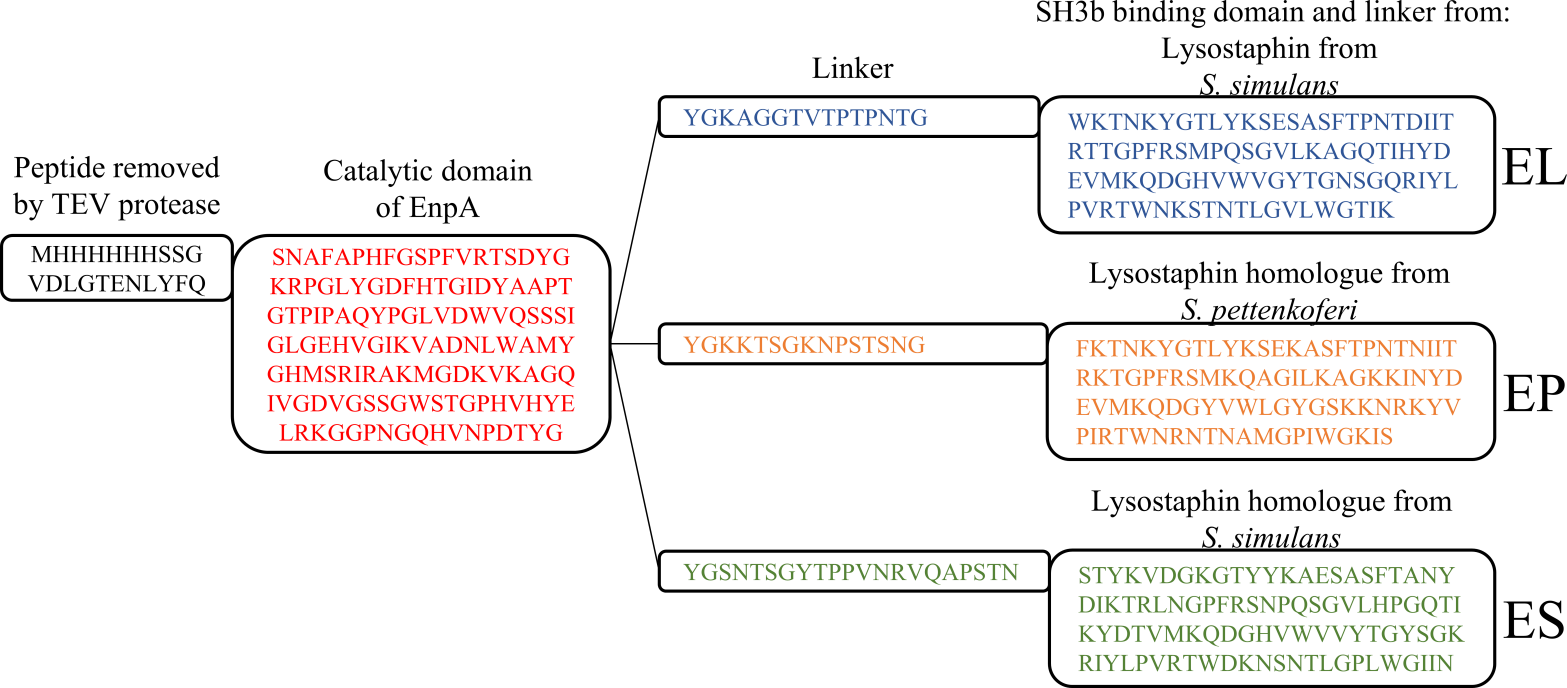
Amino acid sequence of chimeric enzymes EL, EP, and ES. EnpA_CD_ (GenBank ID: AE016830.1, protein ID: AAO81264.1, amino acids 1375–1501), SH3b from lysostaphin produced by *S. simulans* (GenBank ID: U66883.1, protein ID: AAB53783.1, amino acids 387–493), lysostaphin homolog produced by *S. simulans* (GenBank ID: LRQJ01000017.1, protein ID: KXA44996.1, amino acids 310–424) and lysostaphin homolog produced by *Staphylococcus pettenkoferi* (GenBank ID: CP022096.2, protein ID: ASE36562.1, amino acids 341–446). The linker and the SH3b domain are derived from naturally occurring proteins, but the linker fragment has not been modified.

In our previous research, we demonstrated that the fusion of the cell wall-binding domain (SH3b) with the LytM catalytic domain enables chimeric enzyme to maintain its activity even under higher ionic strength (15). To expand the activity of yet another M23 catalytic domain of EnpA_CD_ to wide range of conditions, we applied the same approach. Three chimeric enzymes were engineered by fusing various SH3b domains and linker which occur naturally with this domains, to EnpA_CD_ (GenBank ID: AE016830.1, protein ID: AAO81264.1, amino acids 1375–1501). The selected binding domains were sourced from lysostaphin, produced by *Staphylococcus simulans* (GenBank ID: U66883.1, protein ID: AAB53783.1, amino acids 387–493), and two other lysostaphin homologs produced by *S. simulans* (GenBank ID: LRQJ01000017.1, protein ID: KXA44996.1, amino acids 310–424) and *Staphylococcus pettenkoferi* (GenBank ID: CP022096.2, protein ID: ASE36562.1, amino acids 341–446) (Fig. 1). These three SH3b domains share 44% amino acid sequence identity within their 106 aa-long fragments but differ in pI and net charge (see Table S1). The chimeric enzymes derived from EnpA_CD_ with the attached binding domains from lysostaphin, and M23 peptidases from *S. pettenkoferi*, and *S. simulans* are abbreviated as EL, EP, and ES, respectively. EnpA_CD_ and chimeras were produced with a HisTag, which was cut-off by Tobacco Etch Virus protease, and as a result, three amino acids have been added to the initial sequence senine, asparagine, alanine (SNA). The molecular weight of each purified chimeric enzyme was verified using mass spectrometry (Fig. S1).

### Activity under different ionic strength and pH values

**FIG 2:**
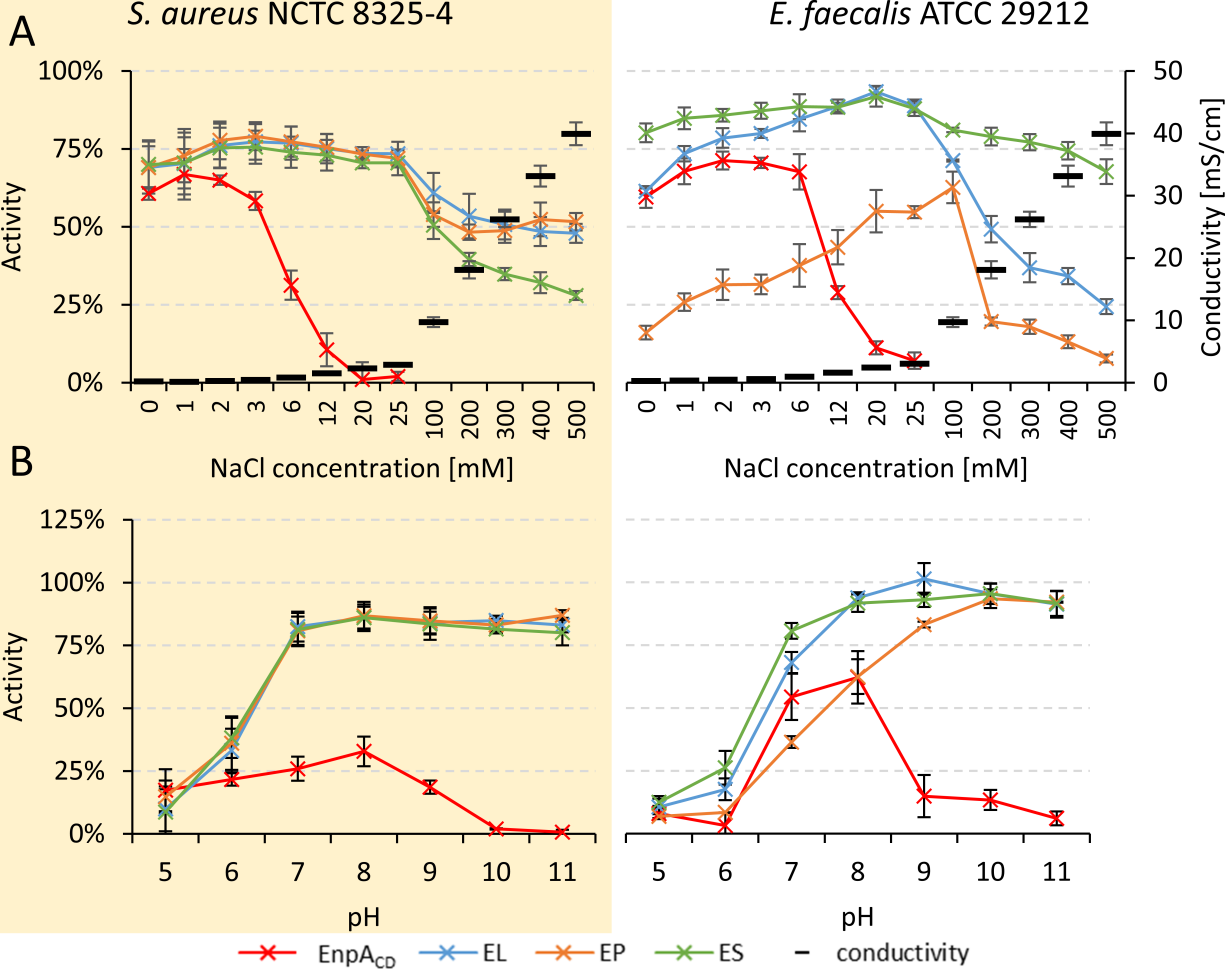
The effect of conductivity (**A**) and pH values (**B**) on EnpA_CD_ and chimeric enzymes activities. *S. aureus* NCTC 8325–4 and *E. faecalis* ATCC 29212 were incubated with 500 nM enzymes in various conductivity (0.1–39.9 mS/cm, pH 8.0) and pH conditions (constant conductivity 0.5 mS/cm) for 1 h at room temperature. The activity was calculated as a percentage of the reduction of the initial OD_620_. The results were normalized to the negative control without added enzyme and presented as mean values and standard deviation derived from at least three technical and biological replications.

To determine the optimal ionic strength for lytic activity, we tested single catalytic domain of EnpA_CD_ and compared to the activity of the chimeras using a turbidity reduction assay against *S. aureus* National Collection of Type Cultures (NCTC) 8325–4 and *E. faecalis* American Type Culture Collection (ATCC) 29212 in 50 mM glycine buffer at pH 8.0, supplemented with various concentrations of NaCl ranging from 0 to 500 mM (0.3–39.9 mS/cm). On the other hand, buffers with conductivity of 0.5 mS/cm were used to determine the optimal pH for enzyme activity by appropriate dilution of the buffering solution with water (Fig. 2). The lytic activity of single domain EnpA_CD_ was hindered by the presence of 6 mM NaCl, being ceased entirely at 25 mM NaCl for both tested bacterial strains. Fusion of the SH3b domains sustained the lytic activity of all three chimeras, EL, EP, and ES, across the entire range of tested ionic strength conditions (Fig. 2A); however, their efficacy declined beyond 25 mM NaCl. Notably, chimeric enzymes’ sensitivity to ionic conditions was clearly depended on the bacterial strain tested. While all three chimeras responded similarly to increasing salt concentrations if *S. aureus* was applied as a substrate, distinct differences in activity were observed among enzymes, when *E. faecalis* cell were investigated. In the latter experiment, ES chimera maintained stable high efficiency across all tested conditions (0–500 mM NaCl), whereas the activity of the EL started to decrease at 25 mM NaCl and continued to drop gradually along with increasing concentration of NaCl. The EP chimera, however, displayed the lowest activity among tested enzymes and exhibited optimal lytic efficacy in the presence of 100 mM NaCl. In summary, the addition of SH3b domains expanded the range of ionic strength permissible for EnpA_CD_ activity.

While the isolated catalytic domain showed activity within a narrow range of pH values, with an optimum pH between 7 and 8, all chimeric enzymes demonstrated lytic activity across a much broader pH range (7–11) (Fig. 2B). The remarkably high activity of all chimeric enzymes under basic conditions is particularly noteworthy, especially considering the complete inhibition of EnpA_CD_ performance in solution of pH above 10 (in *S. aureus* tests) and 9 (in *E. faecalis* experiment). In conclusion, fusion of the cell wall-binding domain with catalytic domain not only broadened tolerance to pH conditions but also enhanced the activity of the chimeric enzymes while compared to the parental enzyme.

### Elimination of bacteria under low and high ionic strength

**FIG 3:**
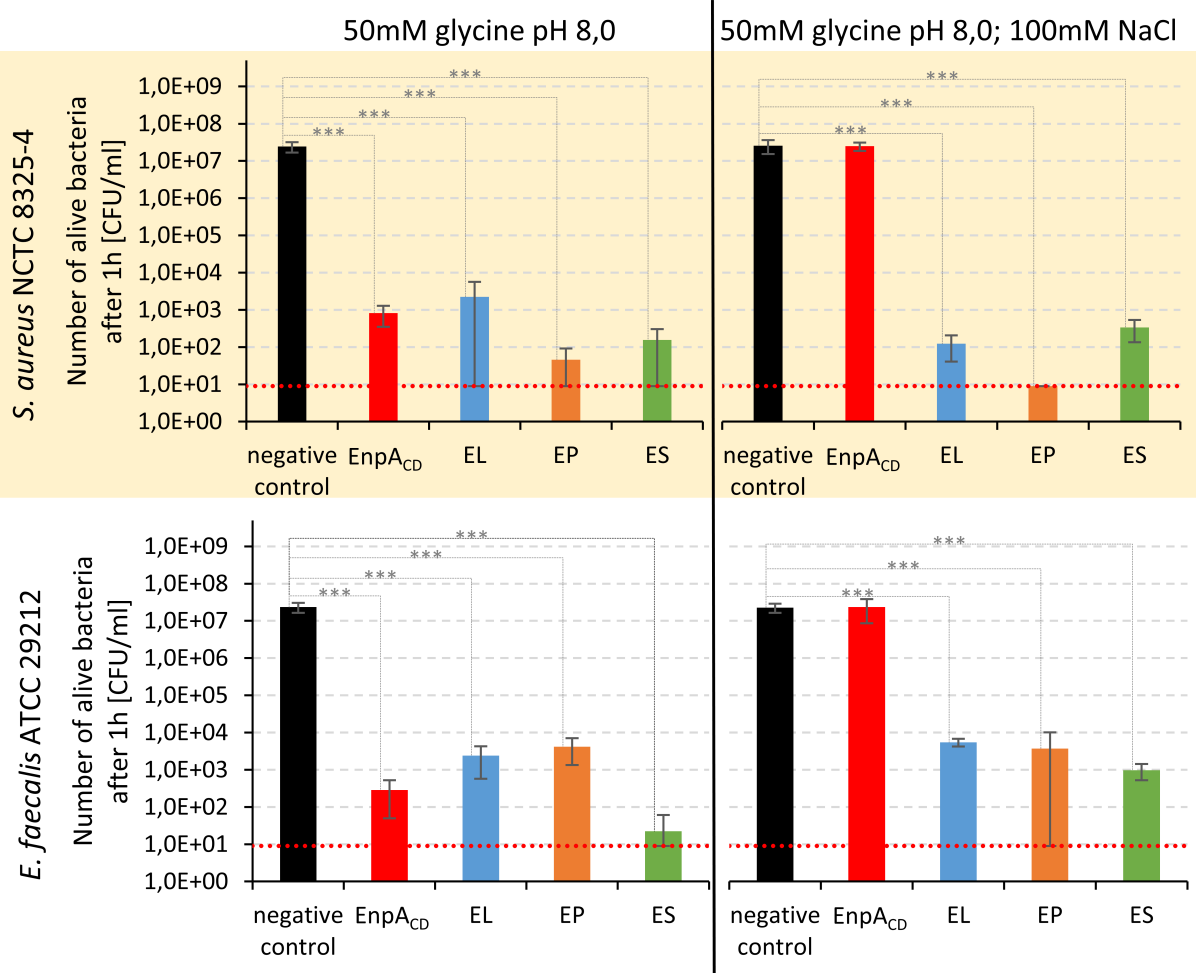
Bacteriolytic potential of studied enzymes in buffer with low (no NaCl) and high (100 mM NaCl) ionic strength against *S. aureus* NCTC 8325–4 and *E. faecalis* ATCC 29212 incubated with 500 nM enzymes for 1 h at room temperature. The number of recovered cells is presented on logarithmic OY axis scale (mean values and standard deviation from three biological replicates). The dashed red line indicates the detection limit of the method. Statistical analysis - one-way analysis of variation (ANOVA) with post-hoc Scheffé multiple comparison test (α = 0.05, *P* value < 0.05 - *, <0.01 -**, <0.001 - ***).

Given that turbidity reduction may not always directly reflect the actual efficiency of enzymes in eliminating bacterial cells, we conducted additional assessments by monitoring the survival of cells after enzyme treatment. The initial count of *S. aureus* NCTC 8325–4 and *E. faecalis* ATCC 29,212 cells was approximately 2.0 × 10^7^ CFU/mL, and the surviving bacterial count was determined after 1 h of treatment with 500 nM enzymes at room temperature (Fig. 3). Under conditions of low ionic strength (50 mM glycine buffer without salt), both EnpA_CD_ and all chimeras exhibited remarkably high activity against both strains. The bacterial cell count decreased from over 10^7^ CFU/mL by at least four logs, with the most significant reduction being six logs. As anticipated, EnpA_CD_ activity diminished under conditions of higher ionic strength (50 mM glycine buffer with 100 mM NaCl), whereas the chimeric enzymes maintained their effectiveness. Statistical analysis revealed no significant differences in activity among the chimeras.

### Specificity against different bacterial strains

**FIG 4:**
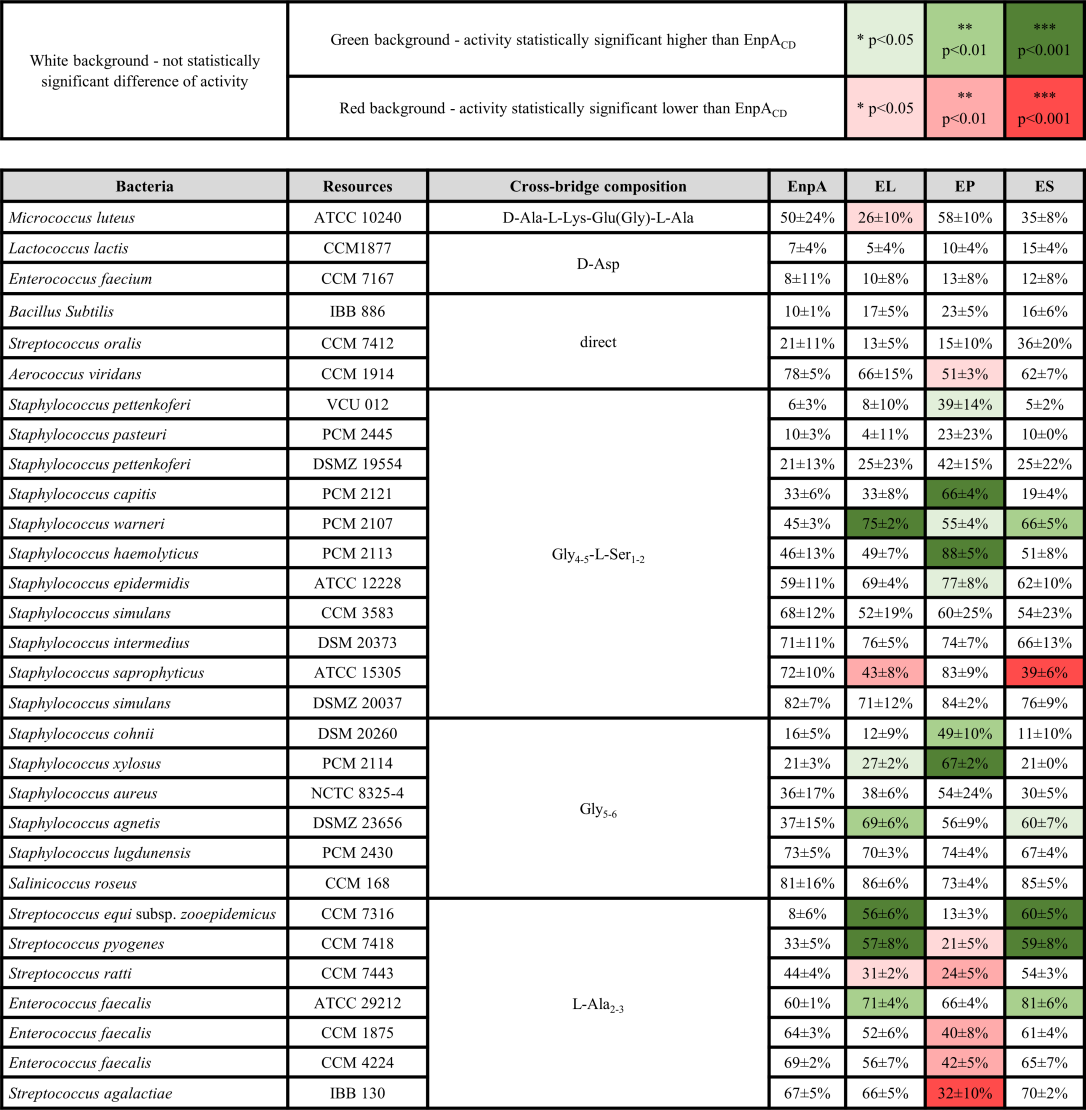
Comparison of the bacteriolytic potential of EnpA_CD_ (tested at low ionic strength) and chimeras (tested at high ionic strength) against different bacterial strains determined by a turbidity reduction assay. The bacteriolytic potential was calculated as the reduction at initial OD_620_ expressed as a percentage of the control (no enzyme) after 1 h of incubation at room temperature. The table shows the mean value and standard deviation from at least three technical and biological replications. (*P* value < 0.05 - *, <0.01 -**, <0.001 - ***, one-way ANOVA with post-hoc Scheffé multiple comparison test).

The specificity of EnpA_CD_ was previously assessed across 32 bacterial strains with diverse PG structures and compositions (10). To investigate the role of the PG type in enzyme specificity, bacterial strains with diverse cross-bridge compositions were selected for the experiment. Furthermore, to evaluate how the addition of binding domains can expand enzymes tolerance to ionic strength, activity tests were conducted and under high-ionic strength conditions for chimeric enzymes (50 mM glycine buffer with 100 mM NaCl) (Fig. 4). In many cases, the chimeric enzymes exhibited higher activity levels against particular bacterial species compared to EnpA_CD_ alone. For instance, the EP chimera displayed activity against *S. pettenkoferi* VCU012, whereas the other enzymes remained inactive. However, with *Streptococcus equi* subsp. *zooepidemicus*, only the EL and ES enzymes demonstrated high activity. In a few cases, the activity of the chimeric enzymes was significantly lower than that observed for parental catalytic domain EnpA_CD_, for example, in tests of ES with *Staphylococcus saprophyticus* and EP with *Streptococcus agalactiae* just to mention the most spectacular (please see Fig. 4).

Bacteria with D-Asp in the PG cross-bridge were least susceptible to all enzymes among tested strains. For bacteria with direct cross-bridges, no significant differences were observed, apart from the EP chimera that displayed reduced activity against *Aerococcus viridans*. The effects were diverse for strains with glycine-serine cross-bridges. The EP chimera exhibited notably higher activity against *Staphylococcus capitis*, *Staphylococcus epidermidis*, *Staphylococcus haemolyticus*, and *S. pettenkoferi* VCU012. In contrast, the EL and ES chimeras demonstrated increased activity against *Staphylococcus warneri* but lower activity against *Staphylococcus saprophyticus*. In the case of bacteria with polyglycine cross-bridges, the activity of all chimeric enzymes matched or surpassed that of EnpA_CD_. Notably, the chimeras exhibited significantly higher activity, especially against strains such as *Staphylococcus cohnii*, *Staphylococcus xylosus*, or *Staphylococcus agnetis*.

The fusion of the SH3b domain yielded the most diverse effects in bacteria with L-Ala in the cross-bridge. For instance, the EL and ES chimeras exhibited high activity against *Streptococcus equi* subsp. *zooepidemicus* and *Streptococcus pyogenes*. In contrast, the EP chimera demonstrated lower activity against most species within this group that were tested.

In accordance with the results obtained, several general conclusions can be drawn: (i) In the majority of cases, the activity level of the generated chimeras in a high ionic strength buffer was either higher or maintained at the same level as the separate catalytic domain in a low ionic strength buffer; (ii) The activity level of the chimera against bacteria with the same murein type varied widely, indicating that the specificity of the chimeric enzymes was influenced by other features of the bacterial cell wall; (iii) In most instances, the EL and ES chimeras displayed similar effects, which were typically opposite to the effects observed for the EP chimera.

### Inhibition of enzyme activity

Bacteriolytic enzymes intended for practical use must exhibit high tolerance to diverse environmental conditions. Moreover, understanding methods for enzyme deactivation is crucial. While the addition of ion chelators is commonly employed to inhibit enzymes with metal ions as cofactors, thermal deactivation represents a more universal approach.

**FIG 5:**
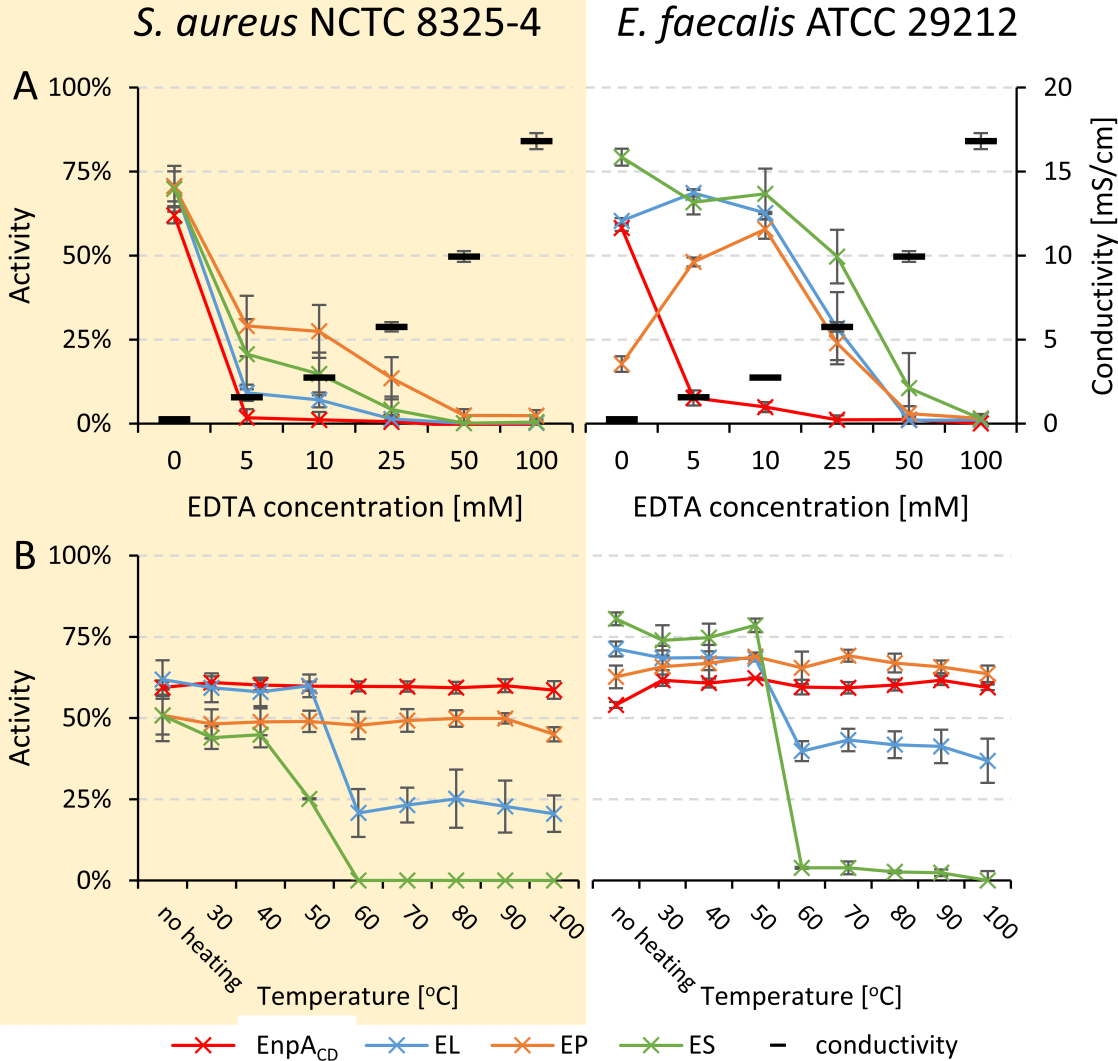
Effect of EDTA (**A**) and heating (**B**) on the activity of chimeras. *S. aureus* NCTC 8325–4 and *E. faecalis* ATCC 29212 were incubated with 500 nM enzymes in 50 mM glycine buffer, pH 8.0 (additionally supplemented with 100 mM NaCl for chimeric enzymes) with increasing concentration of EDTA (0–100 mM, (**A**) or with preheated enzymes (30–100°C, **(B**). The activity is presented as the percentage of the reduction in initial OD_620_ normalized to negative control (no added enzyme). Results are shown as mean value with standard deviation from three technical and biological replications.

In tests conducted with *S. aureus* and *E. faecalis*, EnpA_CD_ lost its activity in the presence of 5 mM EDTA, while the chimeras maintained their activity even at higher concentrations of EDTA. Moreover, the chimeric enzymes exhibited increased sensitivity to EDTA when tested against *S. aureus*. Surprisingly, when treating *E. faecalis*, the EP chimera displayed optimal activity in the presence of 10 mM EDTA (Fig. 5A). Notably, to completely block the activity of chimeras, a very high concentration of EDTA, up to 100 mM, is required.

These observations highlight the differential response of EnpA_CD_ and chimeric enzymes to EDTA, indicating variations in their dependence on metal ions for catalytic activity. Furthermore, the chimeras’ ability to maintain activity at higher EDTA concentrations underscores their robustness and potential utility in practical applications.

The thermostability of the enzymes was assessed by subjecting them to heating for 10 min at temperatures ranging up to 100°C. Results indicated that both EnpA_CD_ and the EP chimera retained their activity even after treatment at the highest temperature. However, the EL chimera exhibited partial inactivation, while the ES chimera was completely inactivated at temperatures exceeding 60°C (Fig. 5B). These findings suggest differential thermal stability among the tested enzymes, with EnpA_CD_ and the EP chimera demonstrating greater resistance to high temperatures compared to the EL and ES chimeras. Understanding the thermostability profiles of these enzymes is crucial for their practical application in various environments and conditions.

### Activity in serum

**FIG 6:**
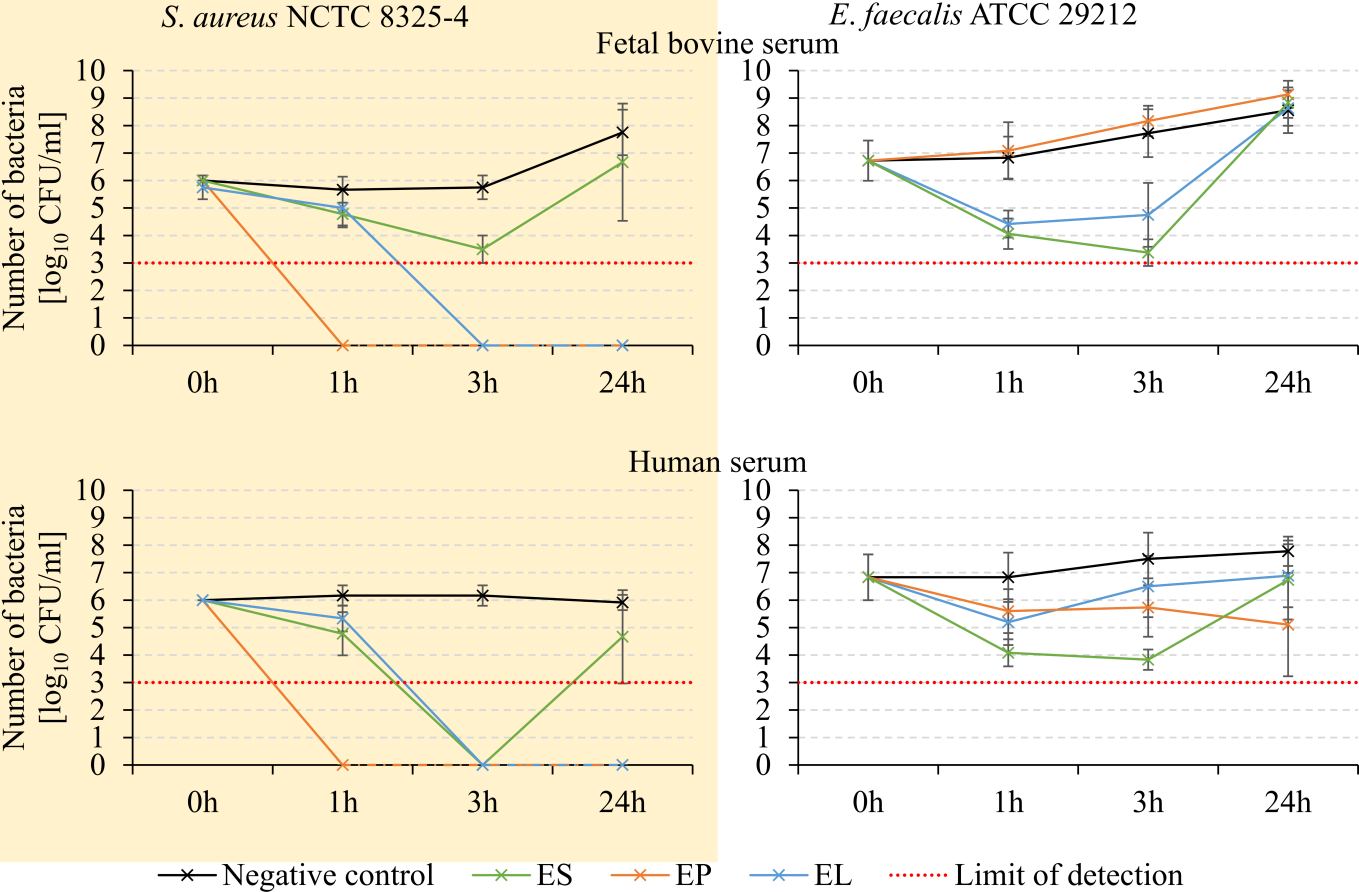
Activity of chimeric enzymes in fetal bovine and human serum. *S. aureus* NCTC 8325–4 and *E. faecalis* ATCC 29212 were incubated in serum with 500 nM enzymes at room temperature for 1, 3, and 24 h. Results are presented as number or recovered bacterial cells (log_10_ CFU/mL) and expressed as mean value with standard deviation from at least three technical and biological replications. The dashed red line indicates the detection limit of the method.

Despite the efficient elimination of bacteria by chimeric enzymes in environments with higher ionic strength, their activity may not be assured under physiological conditions such as serum. The presence of various ions, lipids, and proteins in serum could potentially hinder enzymatic activity. Therefore, the bacteriolytic properties of the chimeras were evaluated against *S. aureus* and *E. faecalis* in the presence of human and fetal bovine serum (FBS) (Fig. 6).

All chimeric enzymes maintained their activity in both human and bovine serum against *S. aureus*. The EP chimera completely eradicated *S. aureus* after 1 h, while for EL and ES, optimal effects were observed after 3 h. However, in the case of ES, regrowth of bacterial cultures was observed after 24 h.

The activity of all chimeras against *E. faecalis* was generally lower compared to *S. aureus*. The ES chimera yielded the best results in this case, although recovery of bacterial cell growth was observed after 24 h in both sera. The EL chimera performed better in FBS than in human serum, where partial regrowth of bacterial cells was observed after 3 h. In contrast, the EP chimera did not exhibit any activity in FBS, while its antibacterial efficiency in human serum, albeit limited, was sustained throughout the experiment.

In summary, in serum environments, the chimeras demonstrate higher activity against *S. aureus*, than *E. faecalis*, with the EL and EP successfully eliminating all bacterial cells, as evidenced by the absence of regrowth after 24 h. *E. faecalis* bacteria, although reduced initially by the enzymes, are not eradicated completely under the tested conditions.

## DISCUSSION

The modular architecture of PGHs offers an opportunity to create chimeric enzymes by recombining individual domains from different proteins (16, 17). This approach can serve various purposes, such as increasing bacteriolytic activity, broadening the spectrum of activity against a greater number of bacterial species, or extending the half-life in the human body (18, 19). Chimeric enzymes can be generated through precise design based on structural and biochemical data or through random shuffling of numerous domains. For instance, the VersaTile high-throughput platform enabled the testing of almost 10,000 chimeras against *A. baumannii*. Remarkably, only five variants were found to be active against four tested strains in 90% human serum (20). This underscores the potential of chimeric enzymes in addressing specific bacterial targets and highlights the importance of innovative approaches in enzyme design and development.

Similar to other isolated catalytic domains of M23 peptidases (15), EnpA_CD_ displayed high activity only under low ionic strength conditions. This phenomenon was also observed in the single catalytic domain of LysK (21), lysostaphin, and mature LytM (15). The primary objective of our protein engineering was to enhance the tolerance of EnpA_CD_ to higher ionic strength by creating chimeric enzymes. These chimeras were designed based on detailed structural and biochemical data, building upon successful strategies employed in previous studies, where fusion of the LytM catalytic domain with the SH3b domain from lysostaphin resulted in chimeric enzymes with extended tolerance for ionic strength and pH values, while maintaining specificity (10, 15, 22). Similarly, the tolerance toward ionic strength and pH values was improved in the EnpA chimeras.

In general, the addition of the SH3b domain did not alter the specificity of the chimeric enzymes compared to EnpA_CD_ indicating that the specificity of the chimera is determined by specificity of the catalytic domain; however, interesting observations were done regarding the role of the binding domains. In several cases, the performance of the chimeric enzymes surpassed that of EnpA_CD_, while in very few cases, it was inferior. Notably, the effects of the EL and ES chimeras were very similar but opposite to those of the EP chimera (Fig. 4).

While our understanding of the domains’ specificity, structure, or binding mechanisms remains limited, and as such, cannot fully elucidate their effects on chimeric enzymes performance, we have made an effort to analyze the relationship between the features of the domains and the performance of the chimeric enzymes. To gain some insights, three-dimensional models of our chimeras were generated using the AlphaFold online tool (Fig. S2) (23). Despite careful analysis and alignment of the generated models with the crystal structure of lysostaphin, drawing relevant conclusions proved challenging. Only subtle differences in loop arrangements were observed, particularly in the most mobile regions. While differences in the mutual arrangement of both domains were noted, they could rise from unstructured character of linkers which are very flexible as demonstrated experimentally for lysostaphin (24). In conclusion, no apparent correlation between the predicted structures of SH3b-binding domains and their demonstrated properties as part of chimeric enzymes could be discerned.

The SH3b domain has been proposed to function as an anchor for the catalytic domain, facilitating enhanced affinity to bacterial cell walls (25). Our previous structural and biochemical studies have shown that the selectivity of the lysostaphin SH3b domain is intricately linked to the architecture of the binding groove, specifically designed to accommodate pentaglycine within staphylococcal PG (26). However, it does not fully account for the enhanced performance of the EL chimera, particularly against bacteria lacking pentaglycine in their PG cross-bridges. One plausible explanation is that this SH3b domains may also interact with the stem peptide of bacterial PG, a conserved feature among various bacterial species. This interaction could contribute to the broader efficacy observed in the EL chimera across different bacterial strains. Recently, we have reported that a domain from *S. pettenkoferi* SpM23B protein demonstrates efficient binding to a wide array of bacterial cells, including Gram-negative species suggesting that SH3b domains might interact with cell wall elements other than PG (27, 28).

Recent findings have highlighted the significant role of electrostatic interactions in mediating enzyme/cell wall interactions, shedding light on the mechanisms underlying bacteriolytic activity (27, 28). Studies have indicated that the positive surface charge of the catalytic domain of PGHs enables them to maintain their bacteriolytic activity even after the removal of the binding domain (29). Moreover, increasing the charge of the binding domain has been associated with enhanced bacteriolytic activity (30). In the case of the generated chimeric enzymes, their features were analyzed using Protein PI online software (31), revealing similar pI values ranging from 9.37 to 9.79. Interestingly, their calculated surface net charge was found to be similar for ES and EL (6.78 and 6.72, respectively), but significantly different for EP (17.67) (Supplemental data Table S1). This variation in surface net charge may contribute to the observed differences in the activities of ES and EL, as well as their opposite effects compared to EP. However, further research is necessary to confirm these conclusions and elucidate the exact mechanisms underlying these observations. One of the challenges in this analysis lies in the heterogeneity of bacterial cell walls and their variability across different species. Understanding the nuances of enzyme-cell wall interactions requires comprehensive investigation and consideration of various factors influencing bacterial susceptibility to enzymatic degradation.

The pH value also affects the surface charge of both proteins and the bacterial cell wall (32, 33). EnpA chimeras exhibit high effectiveness at neutral and alkaline pH but lose their activity at acidic pH due to the absence of direct interactions between protonated histidine and zinc ions at acidic pH (34). A similar phenomenon also occurs with the autolysin AtlE, which becomes inactive in acidic environments below 6.5 (35). In case of AtlA enzyme, this loss of activity could be attributed to protons in the respiratory chain being captured by teichoic acids, leading to local acidification of the environment and inhibition of enzyme activity (36).

The observed differences between chimeras in their response to heat treatment are intriguing. Chimera ES exhibited remarkable resistance to heat and maintained activity even after 10 min of heating at 100°C. In contrast, the EP chimera completely lost activity at 60°C. Interestingly, in a low ionic strength buffer, all chimeras retained their high activity even after heating at 100°C. This suggests that the loss of activity was primarily due to the detrimental effect of the treatment on the SH3b domain rather than the catalytic domain. Additionally, it has been verified that during heating, the chimeras do not break down into single domains, indicating the structural integrity of the chimeric enzymes even under harsh conditions (Fig. S3).

Another interesting observation concerns the increase in EP chimera activity with the increase in EDTA concentration, up to 10 mM and above this concentration, the activity decreases in similar manner like other chimeras. The explanation may be the fact that EP chimer is most active at a particular ionic strength. Also in the case of the EnpA_CD_, its rapid loss of activity is related to the increase in the ionic strength of the solution rather than the action of EDTA (Fig. 2A; Fig. 5A results for *E. faecalis*).

By fusing various cell wall-binding domains to EnpA M23 catalytic domain, we have generated chimeric enzymes with potent bacteriolytic activity expanded to physiological conditions. The development of PGHs as therapeutic agents represents a promising alternative strategy for addressing AMR. PGHs have shown efficacy in reconstituting the balance of skin microflora in various skin disorders, and they are being integrated into dermocosmetics by companies like Micreos. Moreover, PGHs are undergoing clinical trials for systemic treatment of conditions like bacteremia and *S. aureus* endocarditis. The engineered chimeras discussed in our research were specifically designed to function effectively under conditions of high ionic strength and have demonstrated activity in both human and bovine serum. As a result, these chimeras hold significant potential as novel agents against polymicrobial infections caused by different pathogenic bacteria, including *S. aureus* and *E. faecalis*. This versatility and efficacy make them promising candidates for addressing antimicrobial challenges in both topical and systemic applications.

## MATERIALS AND METHODS

### Engineering of EnpA_CD_ and chimeric enzymes

The Polymerase Incomplete Primer Extension (PIPE) cloning method was used to prepare the EnpA_CD_ and chimeras (37). For this purpose, vector (EnpA_CD_ in pMCSG7) (38) and insert amplification (linker and SH3b domain) were prepared using specific primers (Table 1). The resulting construct contained the following components (from the N-terminus): HisTag, Tobacco Etch Virus (TEV) protease cleavage site, EnpA catalytic domain and for chimeras additional SH3b domain with its linker and stop codon. After TEV treatment, three amino acids were added to the N-terminus (serine, asparagine and alanine). The PCR program: 10 µL 5× Phusion Hydrofluoric acid(HF) buffer, 1 µL 10 mM dNTP, 0.5 µM (final concentration) of forward and reverse primers, 20 ng of DNA matrix, and 0.5 µL Phusion High-Fidelity DNA polymerase (Thermo Fisher Scientific). PCR program: 98°C – 30 s, (98°C – 7 s, 55°C – 20 s, 72°C – 90 s) x35 cycles, 72°C – 300 s, 18°C – hold. The PCR products were treated with DpnI (EureX) for 1 h, mixed together in a 1:3 molar vector:insert ratio and heated to 98°C for 5 min to hybridize them. The obtained constructs were sequenced.

**TABLE 1:** Primers’ sequences used for EnpA_CD_ and chimeras cloning

		5′- > 3′ sequence
Vector	Forward	ATAAGTATCAGGATTTACGTGTTGGCCATTTGGTCCGCC
	Reverse	TAAGGCGATACCATAAATTCGAGCTCCGTCGACAAGCTTGCG
EnpA_CD_	Forward	TACTTCCAATCCAATGCCTTTGCTGCACATTTTGGATCACCATTGTT
	Reverse	TTATCCACTTCCAATGTTAGCCATAAGTATCAGGATTTACGTGTTGG
Insert SH3b from Lysostaphin	Forward	AATCCTGATACTTATGGATATGGAAAAGCAGGTGGTACAGTA
	Reverse	TATGGTATCGCCTTACTTTATAGTTCCCCAAAGAACACCTAA
Insert from SpM23_B	Forward	AATCCTGATACTTATGGTTATGGTAAAAAAACTAGTGGTAAG
	Reverse	TATGGTATCGCCTTAACTAATTTTCCCCCAAATTGGTCCCAT
Insert form LssR_M	Forward	AATCCTGATACTTATGGATATGGTAGTAATACTTCTGGTTAT
	Reverse	TATGGTATCGCCTTAATTAATAATTCCCCATAACGGGCCTAA

### Protein expression and purification

Sequence-confirmed plasmids were used to transform *Escherichia coli* BL21 (DE3). Protein expression was carried out in autoinduction medium LB (AIM-LB, Formedium UK) with ampicillin at 25°C overnight with shaking at 180 revolutions per minute(RPM). Bacteria with overexpressed protein were suspended in 50 mM HEPES pH 8.0, 1 M NaCl, 10% glycerol, and 20 mM imidazole (buffer A). The sample was sonicated on ice for 5 min (cycle: 15 s of work, 60 s of rest) and centrifuged (20000 relative centrifugal force (RCF), 30 min, 4°C), and the supernatant with soluble protein was applied to a HisTrap fast flow (FF) 1 mL column (Cytiva). After washing the column with buffer A, the recombinant protein was eluted with 20 mM HEPES pH 8.0, 0.4 M NaCl, 10% glycerol, and 500 mM imidazole and dialyzed overnight in the presence of TEV protease at room temperature in 20 mM HEPES pH 8.0, 0.4 M NaCl, and 10% glycerol (buffer B). The protein solution was reapplied to the HisTrap FF 1 mL column, and the enzyme without HisTag was collected in flow through. Size exclusion chromatography on a HiLoad 16/600 Superdex 75 pg (Cytiva) column using buffer B was carried out as the last step of protein purification. The pure protein was flash frozen in liquid nitrogen and stored at −80°C. Molecular weight of each purified protein was confirmed by mass spectrometry.

### Bacteria preparation for tests

The bacteria from the glycerol stock were streaked on Tryptone Soya Broth (TSB)-agar plates and grown overnight at 37°C. A single colony was grown overnight in TSB medium at 37°C with shaking at 80 RPM and used as 1% inoculum in fresh TSB medium. The bacteria were grown at 37°C with shaking at 80 RPM and collected after reaching a log phase, OD_620_ of 0.6–0.8 (using TECAN Infinite F50 Plus) by centrifugation (3500 RCF, 10 min, 20°C).

### Enzyme activity—determination of number of survived cells (CFU/mL)

The test was carried out in 50 mM glycine-NaOH pH 8.0 (for EnpA_CD_) and with additional 100 mM NaCl (for chimeras). The pelleted bacteria were suspended in reaction buffer (same as enzyme) to obtain ca. 5.0 × 10^7^ CFU/mL. Then 100 µL of the bacterial suspension was mixed with 100 µL of the enzyme solution (final enzyme concentration 500 nM). After 1 h of incubation at RT, 10-fold serial dilutions were made for each sample. One hundred microliters of each dilution was spread on TSB-agar plates, and after an overnight incubation at 37°C, number of colony was counted. Accordingly, the colony forming unit per milliliter (CFU/mL) was calculated. The limit of detection using this method is 10^1^ CFU/mL.

### Enzyme activity in serum

Tested bacteria and enzymes were suspended in human or bovine serum. The pelleted bacteria were suspended in serum to obtain ca. 5.0 × 10^6^ CFU/mL. Then 100 µL of the bacterial suspension was mixed with 100 µL of the enzyme solution (final enzyme concentration 500 nM). Enzyme activity was determined at time 0 and after 1, 3, and 24 h incubation at room temperature by serial 10-fold dilutions. In each time points, 1 µL drop from each serial dilution was placed on a TSB-agar plate and incubated overnight at 37°C. After this time, it was checked in how many drops from the dilution series, the bacteria had grown, and based on this, the number of bacteria in the orders of magnitude that survived was determined. On the basis of that, the log_10_ CFU/mL was calculated. The limit of detection using this method is 3log_10_ CFU/mL.

### Enzyme activity—turbidity reduction assay

The pelleted bacteria and enzymes were suspended in the same buffer (the compositions of the buffers in the individual tests are described below). In each well of the 96-well plate, 100 µL of the bacterial suspension (OD_620_ during test 1.0) was mixed with 100 µL of the enzyme solution (enzyme concentration during test 500 nM). OD_620_ of the reaction was monitored for 1 h using TECAN Infinite F50 Plus with 2-min intervals. The enzyme activity was determined as a percentage of the reduction of initial OD_620_ and normalized to negative control (without added enzyme).

### Effect of heating on enzyme activity

In the assayenzymes were diluted in 50 mM glycine buffer pH 8.0 with 100 mM NaCl (chimeras) or without salt (EnpA_CD_). Then the enzyme solutions were heated for 10 min in a thermoblock at: 30.0, 40.0, 50.0, 60.0, 70.0, 80.0, 90.0 and 100.0°C. The samples were cooled at room temperature and a turbidity reduction assay was performed as described above. Final enzyme concentration was 500 nM and initial OD_620_ of bacteria was 1.0.

### Dependence of enzymatic activity on ionic strength

The turbidity reduction test was carried out with 500 nM enzymes in 50 mM glycine-NaOH buffer, pH 8.0, with the following final NaCl concentrations: 0, 1.0, 2.0, 3.0, 6.0, 12.0, 25.0, 100.0, 200.0, 300.0, 400.0, and 500.0 mM; these prepared buffer had the following conductivity values given in (mS/cm): 0.3 ± 0.1; 0.4 ± 0.1; 0.5 ± 0.1; 0.6 ± 0.1; 1.0 ± 0.1; 1.6 ± 0.1; 2.4 ± 0.1; 3.0 ± 0.2; 9.7 ± 0.8; 18.1 ± 1.4; 26.2 ± 1.2; 33.1 ± 1.7; 39.9 ± 1.8, respectively. Final enzyme concentration was 500 nM and initial OD_620_ of bacteria was 1.0.

### Dependence of enzymatic activity on pH

The turbidity reduction test was carried out in different buffers: citric pH 5.0 and 6.0, Tris-HCl pH 7.0, 8.0, and 9.0, CAPS pH 10.0 and 11.0. Buffers’ concentration was adjusted (by proper dilution with milliQ water) to have the same conductivity of 0.5 mS/cm. Final enzyme concentration was 500 nM and initial OD_620_ of bacteria was 1.0.

### Dependence of enzymatic activity on different EDTA concentrations

The turbidity reduction test was carried out in 50 mM buffer glycine-NaOH, pH 8.0 with the following EDTA final concentrations: 0, 5.0, 10.0, 25.0, 50.0, 100.0 mM. Final enzymes’ concentration was 500 nM and initial OD_620_ of bacteria was 1.0.

### Statistical analysis

The following method was used for statistical analysis, one-way ANOVA with post-hoc Scheffé multiple comparison test. Significance level used to compute the confidence level was *α* = 0.05.

### Mass spectrometry analysis

The molecular weight measurement was performed on a quadrupole time-of-flight (Q-TOF) Premier spectrometer from Waters company. The deconvolution of the obtained spectra (m/z) was performed using software MassLynx Mass Spectrometry Software from Waters company. The MaxEnt 1 tool was used to calculate the mass. MaxEnt 1 is Essential Maximum Entropy Based Tool for interpreting multiply-charged electrospray data).

## Supplementary Material

Reviewer comments

## SUPPLEMENTAL MATERIAL

Supplemental material spectrum.03546-23-s0001.docx Table S1; Fig. S1 to S3.

OPEN PEER REVIEW reviewer-comments.pdf An accounting of the reviewer comments and feedback.

ASM does not own the copyrights to Supplemental Material that may be linked to, or accessed through, an article. The authors have granted ASM a non-exclusive, world-wide license to publish the Supplemental Material files. Please contact the corresponding author directly for reuse.

## References

[1] Mulani MS, Kamble EE, Kumkar SN, Tawre MS, Pardesi KR. 2019. Emerging strategies to combat ESKAPE pathogens in the era of antimicrobial resistance: a review. Front Microbiol 10:539. doi:10.3389/fmicb.2019.00539 30988669 PMC6452778

[2] Murray CJL, Ikuta KS, Sharara F, Swetschinski L, Robles Aguilar G, Gray A, Han C, Bisignano C, Rao P, Wool E, et al.. 2022. Global burden of bacterial antimicrobial resistance in 2019: a systematic analysis. Lancet 399:629–655. doi:10.1016/S0140-6736(21)02724-0 35065702 PMC8841637

[3] Luepke KH, Suda KJ, Boucher H, Russo RL, Bonney MW, Hunt TD, Mohr JF. 2017. Past, present, and future of antibacterial economics: increasing bacterial resistance, limited antibiotic pipeline, and societal implications. Pharmacotherapy 37:71–84. doi:10.1002/phar.1868 27859453

[4] Sharma AK, Kumar S, K. H, Dhakan DB, Sharma VK. 2016. Prediction of peptidoglycan hydrolases- a new class of antibacterial proteins. BMC Genomics 17. doi:10.1186/s12864-016-2753-8 PMC4882796 27229861

[5] Vollmer W, Blanot D, de Pedro MA. 2008. Peptidoglycan structure and architecture. FEMS Microbiol Rev 32:149–167. doi:10.1111/j.1574-6976.2007.00094.x 18194336

[6] Boneca IG, Xu N, Gage DA, de Jonge BL, Tomasz A. 1997. Structural characterization of an abnormally cross-linked muropeptide dimer that is accumulated in the peptidoglycan of methicillin- and cefotaxime-resistant mutants of Staphylococcus aureus. J Biol Chem 272:29053–29059. doi:10.1074/jbc.272.46.29053 9360979

[7] Yang H, Singh M, Kim SJ, Schaefer J. 2017. Characterization of the tertiary structure of the peptidoglycan of Enterococcus faecalis. Biochim Biophys Acta Biomembr 1859:2171–2180. doi:10.1016/j.bbamem.2017.08.003 28784459 PMC5610627

[8] Vermassen A, Leroy S, Talon R, Provot C, Popowska M, Desvaux M. 2019. Cell wall hydrolases in bacteria: insight on the diversity of cell wall amidases, glycosidases and peptidases toward peptidoglycan. Front Microbiol 10:331. doi:10.3389/fmicb.2019.00331 30873139 PMC6403190

[9] de Roca FR, Duché C, Dong S, Rincé A, Dubost L, Pritchard DG, Baker JR, Arthur M, Mesnage S. 2010. Cleavage specificity of Enterococcus faecalis EnpA (EF1473), a peptidoglycan endopeptidase related to the LytM/lysostaphin family of metallopeptidases. J Mol Biol 398:507–517. doi:10.1016/j.jmb.2010.03.033 20347848

[10] Małecki PH, Mitkowski P, Jagielska E, Trochimiak K, Mesnage S, Sabała I. 2021. Structural characterization of EnpA D,L-endopeptidase from Enterococcus faecalis prophage provides insights into substrate specificity of M23 peptidases. Int J Mol Sci 22:7136. doi:10.3390/ijms22137136 34281200 PMC8269130

[11] Nelson DC, Schmelcher M, Rodriguez-Rubio L, Klumpp J, Pritchard DG, Dong S, Donovan DM. 2012. Chapter 7 - endolysins as antimicrobials, p. 299–365. In Łobocka, M, Szybalski, W (eds.), Advances in virus research. Academic Press. 10.1016/B978-0-12-394438-2.00007-4 22748813

[12] Mesnage S, Dellarole M, Baxter NJ, Rouget J-B, Dimitrov JD, Wang N, Fujimoto Y, Hounslow AM, Lacroix-Desmazes S, Fukase K, Foster SJ, Williamson MP. 2014. Molecular basis for bacterial peptidoglycan recognition by LysM domains. Nat Commun 5:4269. doi:10.1038/ncomms5269 24978025 PMC4083421

[13] Lu JZ, Fujiwara T, Komatsuzawa H, Sugai M, Sakon J. 2006. Cell wall-targeting domain of glycylglycine endopeptidase distinguishes among peptidoglycan cross-bridges. J Biol Chem 281:549–558. doi:10.1074/jbc.M509691200 16257954

[14] Gonzalez-Delgado LS, Walters-Morgan H, Salamaga B, Robertson AJ, Hounslow AM, Jagielska E, Sabała I, Williamson MP, Lovering AL, Mesnage S. 2020. Two-site recognition of Staphylococcus aureus peptidoglycan by lysostaphin SH3b. Nat Chem Biol 16:24–30. doi:10.1038/s41589-019-0393-4 31686030 PMC6920042

[15] Jagielska E, Chojnacka O, Sabała I. 2016. LytM fusion with SH3b-like domain expands its activity to physiological conditions. Microb Drug Resist 22:461–469. doi:10.1089/mdr.2016.0053 27351490 PMC5036312

[16] Yang H, Zhang H, Wang J, Yu J, Wei H. 2017. A novel chimeric lysin with robust antibacterial activity against planktonic and biofilm methicillin-resistant Staphylococcus aureus. Sci Rep 7:1. doi:10.1038/srep40182 28067286 PMC5220359

[17] Vázquez R, Domenech M, Iglesias-Bexiga M, Menéndez M, García P. 2017. Csl2, a novel chimeric bacteriophage lysin to fight infections caused by Streptococcus suis, an emerging zoonotic pathogen. Sci Rep 7:16506. doi:10.1038/s41598-017-16736-0 29184097 PMC5705598

[18] Yang H, Linden SB, Wang J, Yu J, Nelson DC, Wei H. 2015. A chimeolysin with extended-spectrum streptococcal host range found by an induced lysis-based rapid screening method. Sci Rep 5:1. doi:10.1038/srep17257 PMC4660466 26607832

[19] Seijsing J, Sobieraj AM, Keller N, Shen Y, Zinkernagel AS, Loessner MJ, Schmelcher M. 2018. Improved biodistribution and extended serum half-life of a bacteriophage endolysin by albumin binding domain fusion. Front Microbiol 9:2927. doi:10.3389/fmicb.2018.02927 30538696 PMC6277698

[20] Gerstmans H, Grimon D, Gutiérrez D, Lood C, Rodríguez A, van Noort V, Lammertyn J, Lavigne R, Briers Y. 2020. A versaTile-driven platform for rapid hit-to-lead development of engineered lysins. Sci Adv 6:eaaz1136. doi:10.1126/sciadv.aaz1136 32537492 PMC7269649

[21] Becker SC, Swift S, Korobova O, Schischkova N, Kopylov P, Donovan DM, Abaev I. 2015. Lytic activity of the staphylolytic Twort phage endolysin CHAP domain is enhanced by the SH3b cell wall binding domain. FEMS Microbiol Lett 362:1–8. doi:10.1093/femsle/fnu019 PMC4811206 25790497

[22] Sabala I, Jagielska E, Bardelang PT, Czapinska H, Dahms SO, Sharpe JA, James R, Than ME, Thomas NR, Bochtler M. 2014. Crystal structure of the antimicrobial peptidase lysostaphin from Staphylococcus simulans. FEBS J 281:4112–4122. doi:10.1111/febs.12929 25039253 PMC4286107

[23] Jumper J, Evans R, Pritzel A, Green T, Figurnov M, Ronneberger O, Tunyasuvunakool K, Bates R, Žídek A, Potapenko A, et al.. 2021. Highly accurate protein structure prediction with AlphaFold. Nature 596:583–589. doi:10.1038/s41586-021-03819-2 34265844 PMC8371605

[24] Tossavainen H, Raulinaitis V, Kauppinen L, Pentikäinen U, Maaheimo H, Permi P. 2018. Structural and functional insights into lysostaphin-substrate interaction. Front Mol Biosci 5:60. doi:10.3389/fmolb.2018.00060 30018958 PMC6038053

[25] Gründling A, Schneewind O. 2006. Cross-linked peptidoglycan mediates lysostaphin binding to the cell wall envelope of Staphylococcus aureus. J Bacteriol 188:2463–2472. doi:10.1128/JB.188.7.2463-2472.2006 16547033 PMC1428428

[26] Mitkowski P, Jagielska E, Nowak E, Bujnicki JM, Stefaniak F, Niedziałek D, Bochtler M, Sabała I. 2019. Structural bases of peptidoglycan recognition by lysostaphin SH3b domain. Sci Rep 9:5965. doi:10.1038/s41598-019-42435-z 30979923 PMC6461655

[27] Wysocka A, Jagielska E, Łężniak Ł, Sabała I. 2021. Two new M23 peptidoglycan hydrolases with distinct net charge. Front Microbiol 12:719689. doi:10.3389/fmicb.2021.719689 34630350 PMC8498115

[28] Wysocka A, Łężniak Ł, Jagielska E, Sabała I. 2022. Electrostatic interaction with the bacterial cell envelope tunes the lytic activity of two novel peptidoglycan hydrolases. Microbiol Spectr 10:e0045522. doi:10.1128/spectrum.00455-22 35467396 PMC9241647

[29] Low LY, Yang C, Perego M, Osterman A, Liddington R. 2011. Role of net charge on catalytic domain and influence of cell wall binding domain on bactericidal activity, specificity, and host range of phage lysins. J Biol Chem 286:34391–34403. doi:10.1074/jbc.M111.244160 21816821 PMC3190764

[30] Díez-Martínez R, de Paz HD, Bustamante N, García E, Menéndez M, García P. 2013. Improving the lethal effect of Cpl-7, a pneumococcal phage lysozyme with broad bactericidal activity, by inverting the net charge of its cell wall-binding module. Antimicrob Agents Chemother 57:5355–5365. doi:10.1128/AAC.01372-13 23959317 PMC3811316

[31] Protein tool. Available from: https://www.protpi.ch/Calculator/ProteinTool. Retrieved 26 Jun 2023.

[32] Schlag M, Biswas R, Krismer B, Kohler T, Zoll S, Yu W, Schwarz H, Peschel A, Götz F. 2010. Role of staphylococcal wall teichoic acid in targeting the major autolysin Atl. Mol Microbiol 75:864–873. doi:10.1111/j.1365-2958.2009.07007.x 20105277

[33] Harden VP, Harris JO. 1953. The isoelectric point of bacterial cells. J Bacteriol 65:198–202. doi:10.1128/jb.65.2.198-202.1953 13034716 PMC169666

[34] Zhou L, Li S, Su Y, Yi X, Zheng A, Deng F. 2013. Interaction between histidine and Zn(II) metal ions over a wide pH as revealed by solid-state NMR spectroscopy and DFT calculations. J Phys Chem B 117:8954–8965. doi:10.1021/jp4041937 23841698

[35] Lützner N, Pätzold B, Zoll S, Stehle T, Kalbacher H. 2009. Development of a novel fluorescent substrate for autolysin E, a bacterial type II amidase. Biochem Biophys Res Commun 380:554–558. doi:10.1016/j.bbrc.2009.01.140 19284999

[36] Biswas R, Martinez RE, Göhring N, Schlag M, Josten M, Xia G, Hegler F, Gekeler C, Gleske A-K, Götz F, Sahl H-G, Kappler A, Peschel A. 2012. Proton-binding capacity of Staphylococcus aureus wall teichoic acid and its role in controlling autolysin activity. PLoS One 7:e41415. doi:10.1371/journal.pone.0041415 22911791 PMC3402425

[37] Klock HE, Koesema EJ, Knuth MW, Lesley SA. 2008. Combining the polymerase incomplete primer extension method for cloning and mutagenesis with microscreening to accelerate structural genomics efforts. Proteins: Struct Funct Bioinf 71:982–994. doi:10.1002/prot.21786 18004753

[38] Stols L, Gu M, Dieckman L, Raffen R, Collart FR, Donnelly MI. 2002. A new vector for high-throughput, ligation-independent cloning encoding a tobacco etch virus protease cleavage site. Protein Expr Purif 25:8–15. doi:10.1006/prep.2001.1603 12071693

