# Supplementary material for: Engineering of chimeric enzymes with expanded tolerance to ionic strength
Source: Microbiol Spectr. 2024 May 2;12(6):e03546-23. doi: 10.1128/spectrum.03546-23 (PMC11237380; doi:10.1128/spectrum.03546-23)
Supplement: Supplemental material — Table S1; Fig. S1 to S3. [file spectrum.03546-23-s0001.docx]

# Supplementary materials

**Table S1** Basic biochemical characteristics and specificity of enzymes used in these studies. pI and net charge value calculated in Prot pI online tool(31).

|  | Molecular weight  [Da] | pI | | | | Net charge at pH 8.0 | | | | Activity against bacteria with a given peptidoglycan type | References |
| --- | --- | --- | --- | --- | --- | --- | --- | --- | --- | --- | --- |
|  |  | two-domain enzyme | Catalytic domain | Linker | Cell binding domain | two-domain enzyme | Catalytic domain | Linker | Cell binding domain |  |  |
| EnpA_CD_ | 13881 | - | 7.98 |  | - | - | 0.05 | - | - | A3α L-Lys-Gly_5-6_ | (9) |
|  |  |  |  |  |  |  |  |  |  | A3α L-Lys-Gly_2-4_-L-Ser_1-2_-Gly |  |
|  |  |  |  |  |  |  |  |  |  | A3α L-Lys-L-Ala_1-2_ |  |
| Lysostaphin  from *S*. *simulans* | 26701 | 9.42 | 8.92 | 8.50 | 9.72 | 8.71 | 1.89 | 0.21 | 4.96 | A3α L-Lys-Gly_5-6_ | (22) |
| Lysostaphin homologue from *S*. *pettenkoferi* | 27194 | 10.07 | 9.81 | 9.81 | 10.19 | 26.53 | 8.90 | 2.18 | 13.86 | A3α L-Lys-Gly_5-6_ | (28) |
|  |  |  |  |  |  |  |  |  |  | A3α L-Lys-Gly_2-4_-L-Ser_1-2_-Gly |  |
| Lysostaphin homologue from *S*. *simulans* | 27719 | 9.31 | 8.70 | 8.509 | 9.52 | 7.68 | 0.94 | 0.22 | 4.88 | A3α L-Lys-Gly_5-6_ | (not published) |
|  |  |  |  |  |  |  |  |  |  | A3α L-Lys-Gly_2-4_-L-Ser_1-2_-Gly |  |
| EL | 25636 | 9.38 | 7.98 | 8.50 | 9.72 | 6.78 | 0.05 | 0.21 | 4.96 | Same as EnpA_CD_ | This study |
| EP | 25854 | 9.79 | 7.98 | 9.81 | 10.19 | 17.67 | 0.05 | 2.18 | 13.86 | Same as EnpA_CD_ | This study |
| ES | 26676 | 9.29 | 7.98 | 8.51 | 9.52 | 6.72 | 0.05 | 0.22 | 4.88 | Same as EnpA_CD_ | This study |


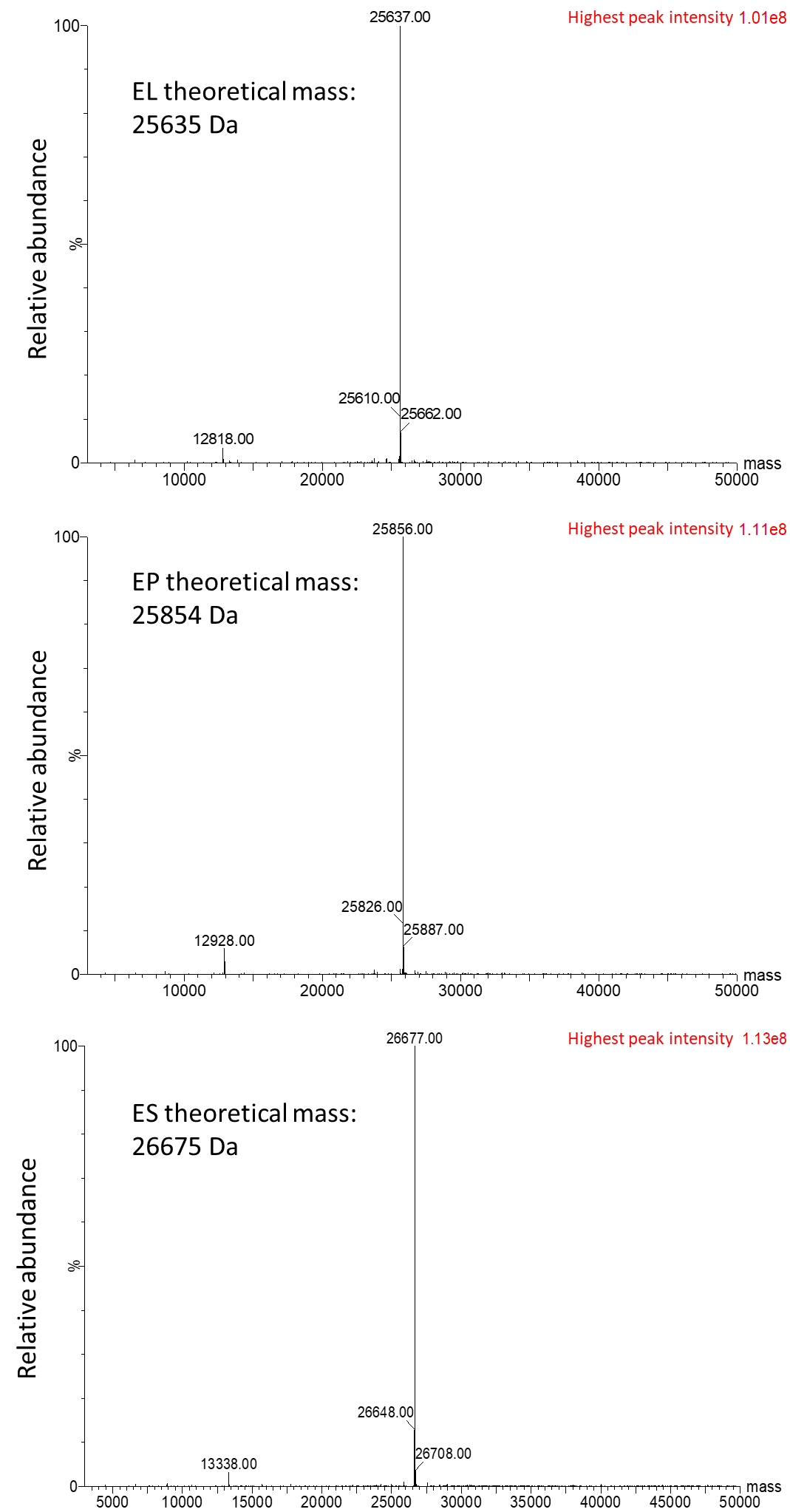


**Figure S1** Deconvoluted mass spectra of the obtained EL, EP and ES chimeras.


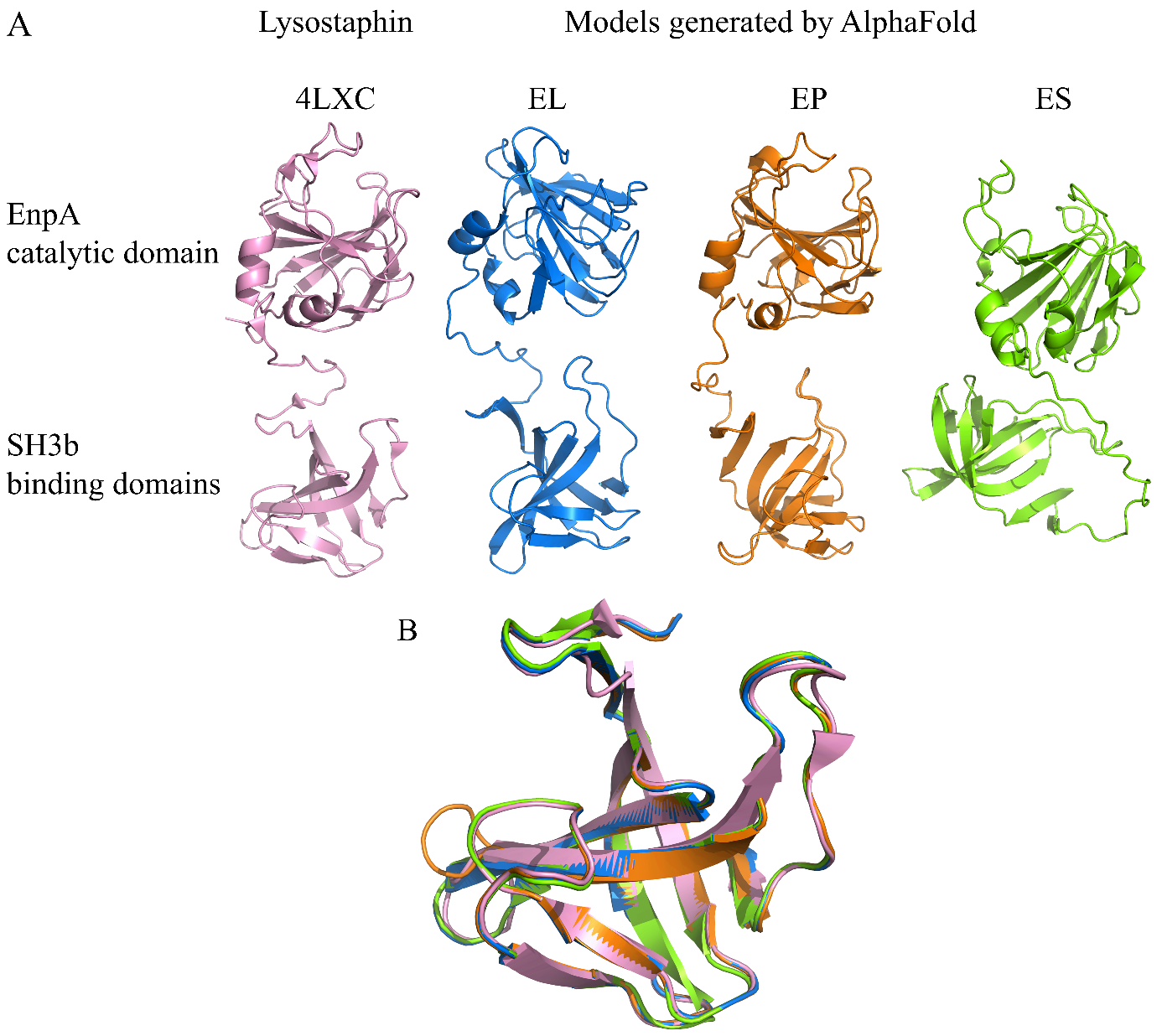


**Figure S2** (A) Three-dimension chimeras’ models generated by AlphaFold (23) and crystallographic structure of lysostaphin (4LXC). (B) Superimposition of the binding domains structures


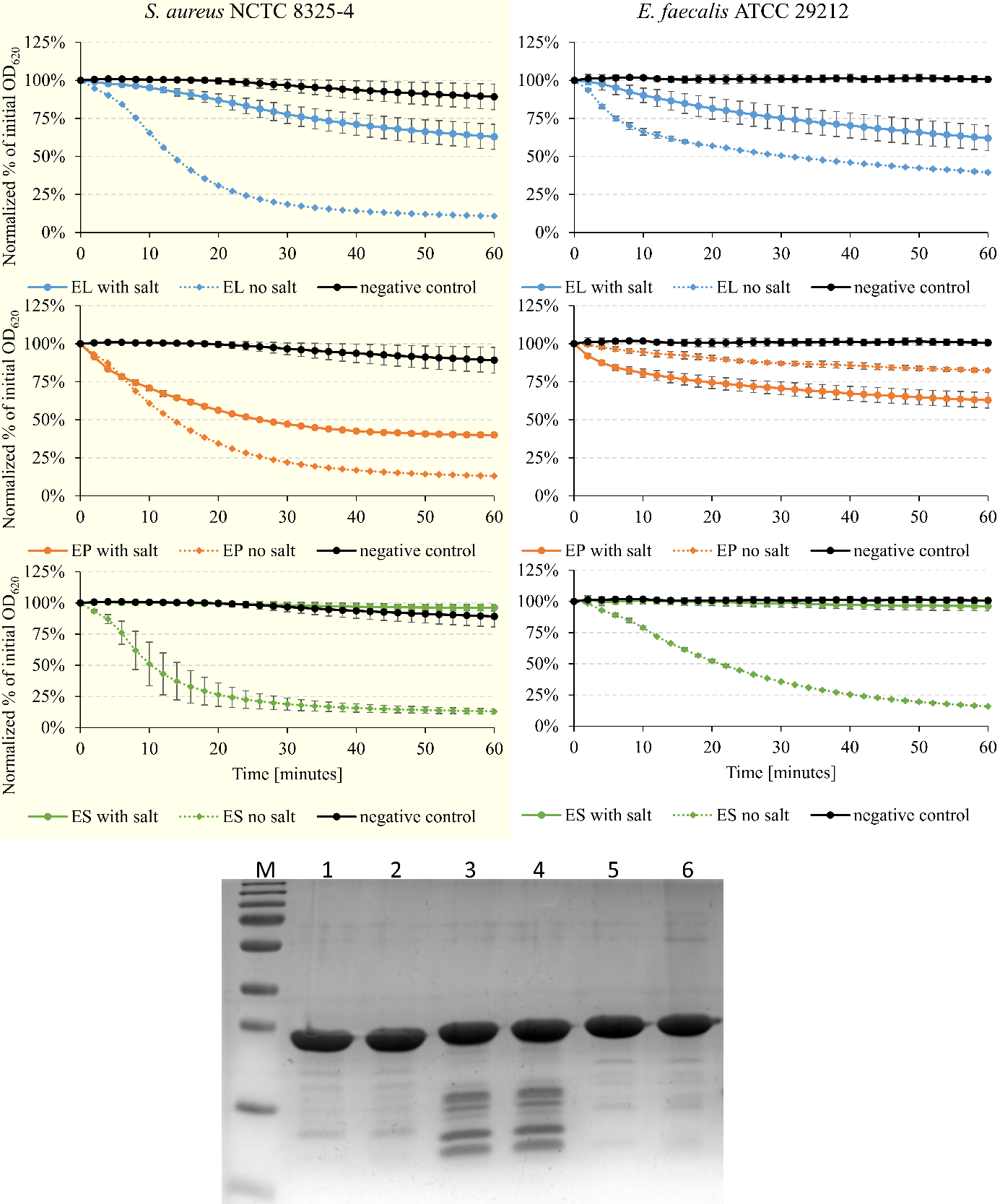


**Figure S3** The Activity of chimeras after heating for 10 minutes at 100 °C against *E*. *faecalis* ATCC 29212 suspension in 50 mM glycine-NaOH pH 8.0 buffer with and without 100 mM NaCl determined by turbidity reduction assay. The graph shows mean value with standard deviation. The tests were carried out in at least three technical and biological replications. Lower panel shows SDS-PAGE of chimeras with and without heating for 10 minutes at 100 °C. Lines: M – molecular weight standard, 1 – EL without heating, 2 – EL after heating, 3 – EP without heating, 4 – EP after heating, 5 - ES without heating, 6 – ES after heating.
